# Descemet's membrane detachment following uneventful phacoemulsification surgeries: A case report

**DOI:** 10.1097/MD.0000000000010444

**Published:** 2018-04-13

**Authors:** Li-yi Chiu, Han-yi Tseng

**Affiliations:** Department of Ophthalmology, Kaohsiung Medical University Hospital, Kaohsiung Medical University, Kaohsiung, Taiwan.

**Keywords:** air tamponade, cataract surgery, Descemet's membrane detachment, phacoemulsification

## Abstract

**Rationale:**

Descemet's membrane detachment (DMD) may occur during or following cataract surgery, causing corneal edema and visual loss1. The incidence of DMD after phacoemulsification surgery is only approximately 0.5%, and mostly surgical-related. Late onset bilateral spontaneous DMD after sequential uneventful cataract surgeries, is even rarer, and may result from not only surgery itself, but also from an underlying anatomic abnormality 2.

**Patient concerns:**

We present a 80 year old female developed bilateral descemet's membrane detachment after sequential uncomplicated cataract surgeries.

**Diagnosis:**

Bilateral Descemet's membrane detachment.

**Interventions:**

One eye (left eye) was treated with intracameral air injection and the fellow eye (right eye) was treated with medical treatment only.

**Outcomes:**

The DMDs were reattached in both eyes after treatment. Surgical intervention accelerated the duration of recovery and there were no significant outcome differences between the right and the left eye.

**Lessons:**

Even if there is a large area of DMD with visual axis involvement, conservative treatment with close observation might still provide a satisfactory result if Descemet's membrane is separated from the posterior corneal stroma by ≤1 mm.

## 1 Instruction

The incidence of Descemet's membrane detachment (DMD) after phacoemulsification surgery is only approximately 0.5%, and mostly surgical related. Late onset bilateral spontaneous DMD after sequential uneventful cataract surgeries, is even rarer, and may result from not only surgery itself, but also from an underlying anatomic abnormality.^[2]^ We present a rare case with bilateral spontaneous DMD after sequential uneventful cataract surgeries. For most case series,^[3–6]^ air tamponade surgery was indicated if a large area of DMD observed. Our case had almost the same course for both eyes although 1 eye was treated with intracameral air injection and the fellow eye was treated with medical treatment only. The DMDs were reattached in both eyes after treatment. We hope this case helps to clarify the roles of surgery and medical management in this clinical setting.

## 2 Case report

**Figure 1:**
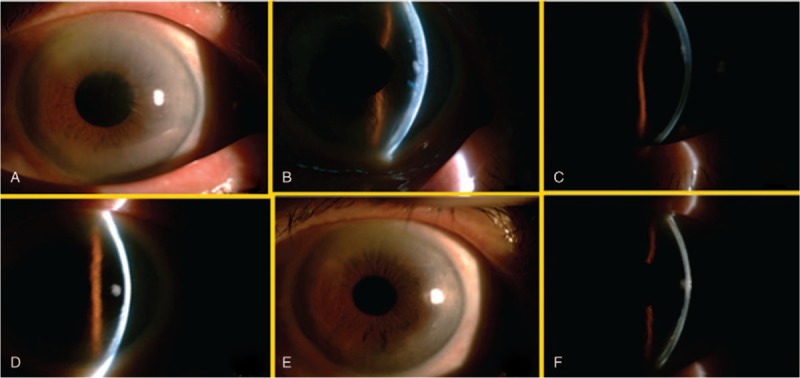
(A and B) Two weeks after cataract surgery (os), prominent DMD noted; (C) 2 weeks after air tamponades; (D) 2 months after air tamponade; (E and F) 6 months after cataract surgery, inferior corneal endothelium scar noted. DMD = Descemet's membrane detachment.

An 80-year-old female without any underlying disease had long-term follow-up in our outpatient department for cataracts of the bilateral eyes. Moderately dense (Grade 3 in The Oxford Clinical Cataract Classification and Grading System) nuclear cataracts were observed bilaterally. Due to progressive visual loss, we performed phacoemulsification cataract surgery for the left eye and the procedure was uneventful. On postoperation days 1 and 7, only mild corneal edema with minimal inflammation was noted. Spontaneous confluent DMD was observed on postoperation day 14 by slit-lamp examination, though not at the surgical site. The DMD was most severe over the superior and mid-periphery area and involved more than 1/2 area of the cornea including the visual axis. Descemet's membrane was separated from the posterior corneal stroma by about 1/2 to 2/3 corneal thickness at the most severe parts. According to Mackool and Holtz^[5]^ classification, it was a planar DMD. The best corrected visual acuity (BCVA) decreased to 0.2 compared to 0.3 preoperation (Fig. 1A and B).

We prescribed preservative-free 0.04% betamethasone eyedrops 8 times daily and 3% hypertonic saline 4 times daily. After 1 week of observation, the condition of DMD with cornea edema was still prominent. We decided to perform anterior chamber tamponade with air under topical anesthesia on postoperation day 21, and finally reattached Descement's membrane.(Fig. 1C and D) The BCVA improved gradually to 0.6, but left a small Descement's membrane scar (Fig. 1E and F).

**Figure 2:**
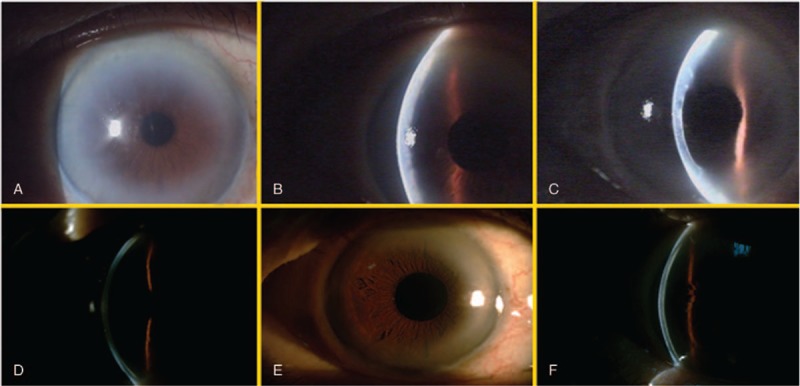
(A and B) One week after cataract surgery (od), significant confluent DMD cornea noted; (C) 2 weeks after medical treatment; (D) 2 months after; (E and F) 6 months after cataract surgery, no scar noted. DMD = Descemet's membrane detachment.

Due to the improvement of the left eye condition, we decided to perform cataract surgery for the right eye 4 months later. Despite a smooth operation, the fellow eye encountered the same situation. On postoperation day 1, only mild corneal edema with minimal inflammation was noted; however, spontaneous significant DMD was observed by slit-lamp examination 5 days after cataract surgery. (Fig. 2A–C). Descemet's membrane was separated from the posterior corneal stroma about 1/2 to 2/3 corneal thickness at the most severe parts with visual axis involvement. According to Mackool and Holtz^[5]^ classification, it was still a planar DMD. We chose conservative treatment with preservative free 0.04% betamethasone with methyl cellulose 8 times daily and 3% hypertonic saline four times daily. Due to the area of DMD and the separation of DMD constantly decreasing during each outpatient follow-up visit, we decided to continue conservative treatment rather than surgical intervention. The detachment totally regressed 2 months after cataract surgery without surgical intervention. (Fig. 2D–F). No scar was left.

The final VA of this patient was 0.4/0.5 compared to initial preoperation VA 0.1/0.2. No recurrent DMD was observed during the following OPD visits.

## 3 Discussion

Unilateral DMD occurs mostly at the time of surgery and is thought to be related to the surgery itself (ex: wound trauma).^[1]^ DMD after uncomplicated cataract surgery is even rarer and may have resulted from an underlying endothelium disorder rather than surgery only.^[3]^ According to Ti and Chee,^[7]^ the main clear corneal incision is the major site of DMD (87.5%), and the risk factors of DMD after phacoemulsification surgery include older age, nuclear sclerosis grade ≥4, pre-existing endothelial disease, and the first postoperation day corneal edema. We should pay more attention to patients with these risk factors. Our case had the risk factor of older age, postoperation day corneal edema and moderate dense cataract.

There are several ways to manage DMD.^[4–8]^ Medical treatment seems to be adequate in many cases and may be an appropriate initial therapy. Air tamponade with room air or SF6 are indicated especially when the DMD area is too large or involving central cornea. If air tamponade fails to reattach Descemet's membrane, further penetrating keratotplasty or endothelial keratoplasty are suggested to restore vision.

Mackool and Holtz^[5]^ classified DMD into planar and nonplanar. Detachments of the DM are classified as planar when there is ≤1 mm separation of the DM from its overlying stroma while nonplanar DMD exceed 1 mm of separation. They concluded that planar detachment has the better prognosis then nonplanar type. In another classification, Samarawickrama et al^[9]^ propose classifying DMD into 2 categories: peripheral and central. In cases where the visual axis is involved, they suggest early intervention with air tamponade.

In a small case series^[10]^ of collected data from PubMed, only 13 patients in the world have been reported to have bilateral DMD after cataract surgeries, most of the cases 20/26 (77%) underwent pneumatic descemetopexy, which permanently reattached the Descemet membrane in 17 cases (85%). Median patient age was 77 years.

After DMD, our case received anterior chamber air tamponade for the left eye and medical treatment for the right eye. According to Mackool and Holtz^[5]^ classification, our case had planar DMD involving bilateral eyes, for which observation may be a treatment choice. However, according to Samarawickrama et al^'^s classification, the area of DMD in our case involved visual axis, and so surgical management was indicated. We performed the air tampoande surgery in the first eye after DMD because we wanted it to recover faster. We decided to observe for the fellow eye because we believe there was a chance of better prognosis without surgical treatment.

The DMDs were reattached in both eyes after treatment. Surgical intervention accelerates recovery duration with no significant outcome differences between the bilateral eyes in our case. We suggest that even DMD with visual axis involvement, if Descemet's membrane is separated from the posterior corneal stroma by ≤1 mm, conservative treatment with close observation might still provide a satisfactory result. For patients with older age, harder lens, and underlying endothelium conditions observed before surgery, we should be more concerned about DMD during and after surgery.

## Author contributions

**Data curation:** Li-yi Chiu, Han-yi Tseng.

**Formal analysis:** Li-yi Chiu, Han-yi Tseng.

**Investigation:** Li-yi Chiu.

**Resources:** Li-yi Chiu.

**Writing – original draft:** Li-yi Chiu.

**Writing – review & editing:** Li-yi Chiu, Han-yi Tseng.

**Methodology:** Han-yi Tseng.
